# THE DEPARTMENT OF VETERANS AFFAIRS HOSPITAL-IN-HOME PROGRAM: A BIRD’S EYE VIEW

**DOI:** 10.1093/geroni/igad104.0537

**Published:** 2023-12-21

**Authors:** William Hung, Orna Intrator, Jennifer Sullivan, Rajesh Makineni, Jiejin Li, Shubing Cai

**Affiliations:** Icahn School of Medicine at Mount Sinai, New York City, New York, United States; Department of Veterans Affairs, Rochester, New York, United States; Center of Innovation in Long Term Services and Supports (LTSS COIN), Providence, Rhode Island, United States; Canandaigua VA Medical center, Canandaigua, New York, United States; Canandaigua VA Medical center, Canandaigua, New York, United States; University of Rochester Medical Center, Rochester, New York, United States

## Abstract

The Department of Veterans Affairs (VA) Hospital-In-Home (HIH) program offers Veterans the option to receive intensive, institutional-level acute services in their homes. This study presents characteristics of Veterans across 11 HIH programs operating between fiscal years 2021 and 2022. VHA administrative data and Medicare claims were linked to identify HIH stays from HIH visits, extending a stay over time as long as visits were no more than 7 days apart, allowing for intervening ED visits. Two types of HIH stays were identified: complementary HIH (admissions from an acute inpatient stay) and substitutive HIH (admissions from all other locations). HIH patients’ sociodemographic characteristics, health conditions, and risk factors are described as well as length of HIH stay (LOS) and discharge destination. We identified 2536 veterans (1,110 complementary and 1,426 substitutive) who had 3,599 HIH stays with 73.9% of Veterans having one stay. Compared to Veterans with complementary HIH, Veterans with substitutive stays were generally younger (67 vs. 72), more likely to be Black (20% vs. 12%), and less sick (probability of 1-year mortality 14% vs 24%). Heart failure, Chronic Obstructive Pulmonary Disease, and COVID were the most frequent admission conditions for both HIH types. Average HIH LOS was 17.3 and 25.8 days for substitutive and complementary models, respectively. About 4% of substitutive and 18% of complementary stays ended in inpatient admission. In the unique VA single-payer, integrated-care system implementations may vary and comparisons of patient characteristics and outcomes can provide the basis for future implementations.

